# Intrapericardial bevacizumab safely and effectively treats malignant pericardial effusion in advanced cancer patients

**DOI:** 10.18632/oncotarget.9420

**Published:** 2016-05-17

**Authors:** Dawei Chen, Yan Zhang, Fang Shi, Hui Zhu, Minghuan Li, Kaijun Chen, Li Kong, Jinming Yu

**Affiliations:** ^1^ Weifang Medical University, Weifang, China; ^2^ Department of Radiation Oncology, Shandong Cancer Hospital affiliated to Shandong University, Jinan, China; ^3^ Shandong Academy of Medical Sciences, Jinan, China; ^4^ Department of Clinical Laboratory, Mengyin People's Hospital, Linyi, China

**Keywords:** bevacizumab, intrapericardial, malignant pericardial effusion, advanced cancer

## Abstract

We evaluated the safety and efficacy of intrapericardial bevacizumab (BEV) for treating symptomatic malignant pericardiac effusion (MPCE) in seven advanced cancer patients. All patients had previously undergone multiple lines of systemic therapy. Each patient received paracentesis and intrapericardial infusions of 100 or 200 mg of BEV every two weeks. Systemic treatments for primary tumors continued for all patients during BEV treatment. Of the seven patients, three achieved a complete response, two achieved a partial response, and two showed no response with regard to MPCE after BEV infusion. The median overall survival time was 168 days (range, 22-224 days). In six of the seven patients, effusion did not recur before death. Toxicity associated with BEV treatment was mild and manageable in all patients. This study provides preliminary evidence that intrapericardial BEV may be an effective and safe treatment for MPCE in patients with advanced cancers.

## INTRODUCTION

Malignant pericardial effusion (MPCE), a common complication that causes refractory cardiac dysfunction and chronic pericardial tamponade, affects up to 15% of advanced cancer patients and shortens their overall survival [1, 2]. MPCE develops rapidly and is life-threatening, requiring prompt and effective salvage therapy [3]. Current clinical treatments for MPCE include indwelling pericardial catheters, fenestrated drainage, and intrapericardial infusion of therapeutic agents [4]. However, the efficacy of these treatments is poor, and effusion eventually reoccurs [5, 6]. Therefore, in order to improve patients' quality of life, more effective therapies for MPCE are necessary.

Recent studies showed that intrapericardial infusion of bevacizumab (BEV) was an effective treatment for malignant ascites and pleural effusion [7, 8, 9, 10]. Here, we examined the effects of BEV in advanced cancer patients with MPCE.

## MATERIALS AND METHODS

### Patients

Seven advanced cancer patients with symptomatic MPCE were included in our retrospective study. All patients received intrapericardial infusion of BEV at Shandong Cancer Hospital between May 2011 and November 2013. Advanced cancer was diagnosed by pathology. MPCE was detected using ultrasonography and computed tomography and confirmed by cytology. The median age of the patients was 45 years (range, 23–75 years). Four patients had stage IV lung cancer, and the remaining three patients had esophageal cancer, malignant mesothelioma of the pleura, and a mediastinal yolk sac tumor. Typical cardiac tamponade symptoms were observed in all patients, including dyspnea in seven patients, stethalgia in six patients, and tachycardia in five patients. All patients had previously undergone multiple lines of systemic therapy. Frequent paracentesis was required in order to relieve cardiac tamponade symptoms. Table 1 summarizes the clinical characteristics of the patients. This study was approved by the Shandong Cancer Hospital Institutional Review Board.

**Table 1:** Clinical characteristics of patients with malignant pericardial effusion

Patient No.	Primary tumor	Gender	Age	Pathological type	Stage	ECOG Score	Pre -systemic therapy	Pre-intrapericardial infusion	Symptoms
1	Lung cancer	M	38	Adenocarcinoma	pT_4_N_1_M_1_	2	Pneumonectomy, chemotherapy, TKI	#IL-2, CDDP	Dyspnea, Stethalgia,
2	Lung cancer	M	60	Adenocarcinoma	cT_4_N_2_M_1_	2	Chemoradiotherapy	CDDP	Dyspnea, Stethalgia, Tachycardia
3	Lung cancer	F	68	Adenocarcinoma	cTXNXM_1_	2	Chemoradiotherapy	-	Dyspnea, Stethalgia, Tachycardia
4	Lung cancer	F	40	Adenocarcinoma	cT_4_N_2_M_1_	3	Chemoradiotherapy, TKI	-	Dyspnea, Stethalgia, Tachycardia
5	Esophageal cancer	M	75	Squamous	pT_3_N_2_M_1_	1	Chemotherapy	#CDDP	Dyspnea
6	MMP	M	45	Epithelial type	cT_4_N3M_1_	4	Chemotherapy, TKI	-	Dyspnea, Stethalgia, Tachycardia
7	Mediastinal tumors	M	23	Yolk sac tumor	cT_4_N_2_M_1_	4	Chemotherapy	#CDDP	Dyspnea, Stethalgia, Tachycardia

Abbreviations: MMP, malignant mesothelioma of pleura; M, male; F, female; ECOG, Eastern ; Cooperative Oncology Group; TKI, tyrosine kinase inhibitor; BEV, bevacizumab; CDDP, cisplatin; IL-2, interleukin-2

### Intrapericardial infusion of BEV

All patients required pericardial catheterization with the aid of ultrasound. Each patient received drainage more than one time to remove pericardial effusions as much as possible. When no obvious effusion remained, patients received an intrapericardial infusion of 100 or 200 mg of BEV diluted in 30 mL of physiological saline. To ensure the uniform distribution of bevacizumab in the pericardial cavity, patients were advised to turn over smoothly every 15 min. This treatment was repeated every two weeks until a response was observed.

### Concurrent systemic therapy

In addition to BEV pericardial infusion, the patients continued to receive systemic therapies to treat their primary tumors. Two patients with confirmed *EGFR* gene mutations received concurrent erlotinib (150 mg every day). Four patients received systemic chemotherapy that included 75 mg/m^2^ cisplatin on days 1, 2, and 3, and either 500 mg/m^2^ pemetrexed on day 1 or 1000 mg/m^2^ gemcitabine on days 1 and 8 (Table 2). All chemotherapy cycles were repeated every 3 weeks for 2-4 cycles. Patients who did not show progression after treatment with the above regimens received pemetrexed or gemcitabine maintenance therapy every 3 weeks until disease progression. One patient with an Eastern Cooperative Oncology Group (ECOG) score of 4 could not tolerate systemic chemotherapy and received supportive treatment exclusively.

**Table 2:** Protocols for intrapericardial infusion of BEV and response to therapy

Patient No.	BEV infusion (mg) × times	Interval times (days)	Concurrent systemic therapy	Response	EFS(days)	Survival(days)
1	100 × 2	14	Erlotinib	CR	196	196
2	200 × 2	14	PEX + CDDP	CR	168	168
3	200 × 2	14	PEX + CDDP	PR	102	102
4	100 × 3	14	Erlotinib	CR	224	224
5	100 × 2	14	GEM+ CDDP	PR	182	182
6	200 × 1	14	GEM+ CDDP	NR	22	22
7	200 × 2	14	-	NR	40	46

Abbreviations: BEV, bevacizumab; CR, complete response; PR, partial response; NR, no response; GEM, gemcitabine; PEX, pemetrexed; CDDP, cisplatin; EFS, effusion-free survival time.

### Evaluation of intrapericardial infusion of BEV

Ultrasonography and computed tomography were used to evaluate the therapeutic efficacy of intrapericardial infusion of BEV for MPCE. According to previous studies [8, 9, 10] and the Response Evaluation Criteria in Solid Tumors (RECIST, version 1.1), therapeutic efficacy of BEV for MPCE was classified as follows: 1) complete response, MPCE completely disappeared within 4 weeks; 2) partial response, MPCE was reduced more than 50% within 4 weeks; or 3) no response, MPCE was reduced by less than 50% or effusion increased. Effusion-free survival time was used to evaluate the duration of the local pericardial response. To assess adverse events, we used the National Cancer Institute Common Terminology Criteria for Adverse Events grading system (CTCAE, version 4.0).

## RESULTS

### Efficacy

**Figure 1:**
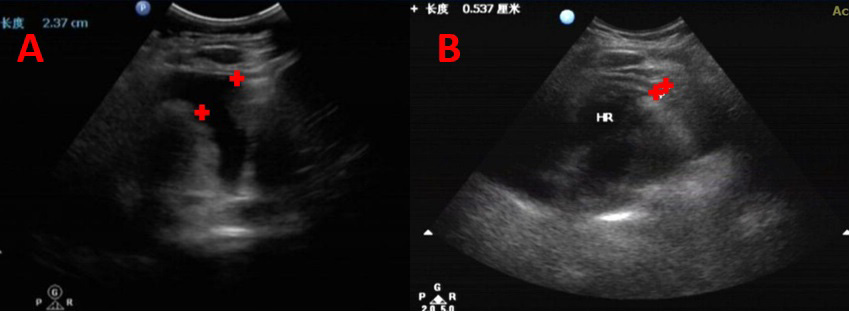
B-ultrasonography images of a 60-year-old man with advanced lung cancer who had recurrent malignant pericardial effusion (MPCE) and who had received multi-line therapy to control MPCE with unsatisfactory outcomes MPCE almost completely disappeared after bevacizumab perfusion until his death. (A) Five days before bevacizumab intrapericardial infusion. (B) Four months after bevacizumab intrapericardial infusion

**Figure 2:**
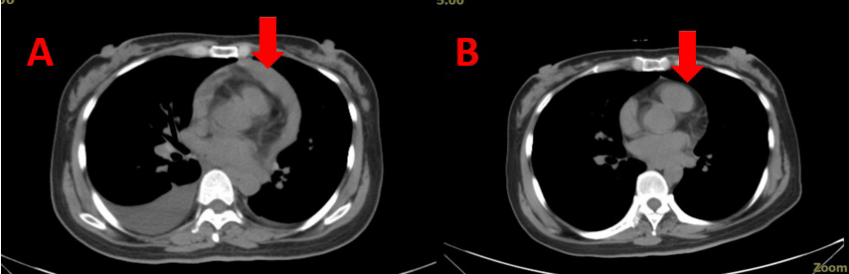
Computed tomography images of a 40-year-old woman with advanced lung cancer who had recurrent malignant pericardial effusion (MPCE) and had received chemoradiotherapy and tyrosine kinase inhibitor therapy without a satisfactory outcome However, bevacizumab infusion was effective. (A) Two days before bevacizumab intrapericardial infusion. (B) Six months after bevacizumab intrapericardial infusion.

One of the seven patients completed one cycle, five patients received two cycles and one patient completed three cycles of pericardiocentesis with BEV infusion. The drainage catheter was successfully removed from all patients. Noticeable remission of cardiac tamponade symptoms was observed in six patients, and typical symptoms such as dyspnea, stethalgia, and tachycardia did not recur during the follow-up period. Three patients achieved complete response (Figures 1 and 2), two patients achieved partial response, and two patients had no response. In six of the patients, effusion did not reoccur before death, and effusion-free survival times ranged from 22 to 224 days (median, 168 days). Only one patient experienced effusion recurrence (Table 2).

### Drug-related adverse events

All drug-related adverse events experienced by patients were mild and manageable (Table 3). The common side effects that accompany systemic chemotherapy, including hematological toxicity, hepatotoxicity, and nephrotoxicity, were not observed. Two patients experienced nausea and vomiting. One patient experienced mild proteinuria related to bevacizumab treatment. None of the patients experienced hypertension. In addition, one patient experienced thrombosis in both legs, accompanied by abnormal clotting, which was resolved by anticoagulation treatment. None of the patients discontinued treatment because of serious adverse events.

**Table 3:** Drug-related adverse events for bevacizumab intrapericardial infusion (CTCAE version 4.0)

Patient No.	Proteinuria	Thrombus	Hypertension	Nausea and vomiting	Hemorrhage	Hematological toxicity	Hepatotoxicity	Nephrotoxicity
1	-	-	-	G1		-	-	-
2	G1	-	-	-		-	-	-
3	-	G2	-	-		-	-	-
4	-	-	-	-		-	-	-
5	-	-	-	-		-	-	-
6	-		-	G2		-	-	-
7	-	-	-	-		-	-	-

Abbreviations: G1 and G2, grade of adverse events in patients according to CTCAE, the National Cancer Institute Common Terminology Criteria for Adverse Event.

## DISCUSSION

MPCE is a common complication in advanced cancer patients and is associated with very poor prognosis, and current treatments for MPCE are not effective [5,6]. Additionally, the mechanisms underlying MPCE are poorly understood. According to previous studies, MPCE usually results from lymphatic obstruction due to the spread of cancer into draining lymph vessels [11, 12, 13, 14]. Angiogenesisis may contribute to the production of malignant effusion [15, 16, 17], and tumors often overexpress vascular endothelial growth factor (VEGF), which is a major pro-angiogenic factor [18]. VEGF can cause tumor vessels to become disorganized, leaky, and tortuous, and can promote the production of malignant effusions in serous cavities such as the pericardial cavity [19, 20, 21].

Because the development of effusion likely results from abnormal vascularity and the permeability of tumors [22, 23, 24, 25, 26], anti-VEGF agents might be effective in treating MPCE. BEV, which blocks the binding of human VEGF to its receptors, is a recombinant humanized monoclonal anti-VEGF antibody [27]. In preclinical studies, BEV inhibited the growth of 13 types of malignant cells [28]. Additionally, it decreased the density, diameter, and permeability of vessels, consequently reducing interstitial fluid pressure [22]. In animal models [22, 23] and clinical studies [7, 8, 9], BEV inhibited the development of malignant effusions in serous cavities more safely and effectively than commonly used cytotoxic agents, such as cisplatin and 5-fluorouracil. There are very few reports investigating intrapericardial BEV as a treatment for MPCE. In a previous exploratory study, we infused BEV into the pericardium of a lung cancer patient with MPCE and achieved complete response, with the patient remaining free of fluid for five months [29]. Given this encouraging result, we repeated this treatment here in seven patients with refractory MPCE to confirm its efficacy. The median survival time of these patients was 168 days (range, 22–224 days). All patients achieved obvious and sustained symptom remission, and six of the seven patients did not suffer from effusion recurrence before death. Similar studies using intrapericardial cisplatin reported median survival times of 84 ± 39 days [30] and 120 ± 71 days (range, 68–268 days) [31]. Our results in this group of patients suggest that intrapericardial BEV may be more effective than other therapies in treating MPCE.

Among the patients in our study, two had point mutations in exon 21 of the *EGFR* gene and developed MPCE during erlotinib treatment. Both of these patients received intrapericardial infusions of BEV while continuing with the initial erlotinib regimen. These patients, who did not receive any additional systemic therapies, had a longer remission period than the other patients. Similar to previous data [32], these results suggest that both EGFR gene mutations and erlotinib efficacy may be associated with better prognoses. Moreover, BEV perfusion might be especially beneficial for MPEC patients who experience disease progression during erlotinib treatment. For lung cancer patients with MPEC and EGFR gene mutations, intrapericardial BEV might therefore be particularly effective.

We found that intrapericardial BEV was a safe and effective treatment for MPCE, and toxicities associated with this treatment were mild and endurable. Maisch et al. [30] and Oida et al. [31] reported nausea in most patients who received cisplatin infusions. In contrast, no hematological toxicity, hepatotoxicity, or nephrotoxicity was observed in any of the patients in our study after BEV treatment. Bilateral thrombosis was observed in one patient who had a history of atherosclerosis and myocardial infarctions and an elevated blood level of D-dimer before treatment, all of which are associated with high risk for bilateral thrombosis. Intravenous infusion of BEV has been shown to cause thrombosis [7, 21], but it remains unclear whether intrapericardial infusion of BEV has the same effect. Although the occurrence of thrombosis was not necessarily due to BEV infusion, patients with abnormal clotting conditions who receive this treatment should be closely monitored.

In previous studies, the doses of BEV used to treat ascites and pleural effusion were 15 mg/kg and 7.5 mg/kg [8, 34]. To date, there is little information regarding the most appropriate dosage of BEV for intrapericardial perfusion. In our study, most of the patients received a 200 mg dose. This dosage was on the high end of the previously investigated dose range (7.5 mg/kg – 15 mg/kg). Additionally, three patients received 100 mg of BEV due to poor ECOG scores (two patients) or advanced age (75 years, one patient). However, this lower dose was still effective in treating MPCE. Because of the small number of patients in our study, we were unable to determine the most appropriate dosage of BEV.

The results of this study provide early evidence that intrapericardial BEV may be an effective and safe treatment for MPCE arising from various malignancies. Further studies are required to investigate the side effects of this treatment and the appropriate BEV dosage. These studies should include larger patient cohorts to allow for appropriate statistical analyses.
